# Influence of Alkaline Reduced Water Supplementation on Glucose and Lipid Metabolism in Non-Diabetic and Diabetic Rats

**DOI:** 10.3390/nu16234082

**Published:** 2024-11-27

**Authors:** Chung-Hsiung Huang, Ying-Chiun Chao, Meng-Tsan Chiang

**Affiliations:** Department of Food Science, National Taiwan Ocean University, Keelung 20224, Taiwan; huangch@mail.ntou.edu.tw (C.-H.H.); m-uly@yahoo.com.tw (Y.-C.C.)

**Keywords:** alkaline reduced water, diabetes, carbohydrate metabolism, lipid metabolism, oxidative damage

## Abstract

Background: With the global increase in metabolic disorders, identifying effective dietary strategies is crucial for enhancing health outcomes. While various health advantages of alkaline reduced water (ARW) have been documented, its specific impacts on glucose and lipid metabolism in both healthy and diabetic conditions are still not well understood. Methods: This study investigates how ARW affects carbohydrate and lipid metabolism in male Wistar rats, which were induced to develop glucose metabolism disorders through subcutaneous injections of nicotinamide and streptozotocin (STZ). The rats were allocated into four groups: one group received distilled water, another ARW, with similar arrangements for both non-diabetic and diabetic rats. Throughout the six-week experiment, the rats had unrestricted access to food and water. At the end of the study, blood and tissue samples were collected post-euthanasia for further analysis. Results: Non-diabetic rats consuming ARW experienced significant decreases in plasma glucose, triglycerides, cholesterol, insulin, leptin, and TBARS levels, along with reduced activities of hepatic hexokinase and intestinal sucrase. Meanwhile, there were increases in hepatic antioxidant enzyme activities, such as glutathione peroxidase and glucose-6-phosphate dehydrogenase, although glutathione levels decreased. In diabetic rats, ARW supplementation notably reduced plasma glucose and the glucose area under the curve, lowered hepatic glucose-6-phosphatase and intestinal disaccharidase activities, and raised hepatic GSH levels. Conclusions: These findings suggest that ARW supplementation significantly enhances glucose and lipid metabolism and boosts antioxidant activity in both non-diabetic and diabetic rats, indicating its potential as a therapeutic aid for managing metabolic disorders.

## 1. Introduction

Diabetes mellitus (DM) is a multifaceted metabolic condition marked by high blood sugar levels, resulting from either inadequate insulin production or diminished insulin sensitivity [1]. This condition can result in significant microvascular and macrovascular complications, especially in type 2 diabetes mellitus (T2DM), where insulin resistance and impaired glucose regulation increase the likelihood of cardiovascular diseases [2]. Hyperglycemia is a significant contributor to atherosclerosis, making effective blood sugar management essential to reducing the risk of cardiovascular diseases, a primary contributor to mortality in T2DM [2]. The oral glucose tolerance test (OGTT) is a vital diagnostic tool for T2DM, assessing the body’s glucose management over time [1]. Important metrics for evaluating metabolic dysfunction include drinking habits, urine output, dietary intake, and body weight [3]. In T2DM, variations in plasma glucose and insulin levels, along with markers such as fructosamine, offer valuable insights into the degree of insulin resistance and the effectiveness of glycemic control [2]. The relationship between hyperglycemia, dyslipidemia, and oxidative stress is crucial for understanding diabetes pathology [2]. High glucose and lipid levels increase reactive oxygen species (ROS) production, exacerbating oxidative stress and cellular damage. Normally, intracellular antioxidative enzymes could neutralize ROS. However, in diabetic conditions, this balance is disrupted, leading to further complications [4].

Researchers often utilize the nicotinamide/streptozotocin (STZ) rat model to replicate the pathophysiology of T2DM. Nicotinamide, in conjunction with STZ, which selectively targets and injures pancreatic β-cells, leads to insulin resistance. It successfully replicates essential characteristics of T2DM, such as β-cell dysfunction, hyperglycemia, and dyslipidemia [5]. A high-fat diet (HFD) significantly contributes to metabolic disorders by promoting hyperglycemia, hyperlipidemia, and oxidative damage. Excessive fat intake leads to insulin resistance and elevated free fatty acids, disrupting glucose metabolism [6]. Additionally, an HFD causes lipid imbalances, increasing triglycerides and low-density lipoprotein (LDL) cholesterol, which further exacerbates insulin resistance and cardiovascular risk [7]. An HFD also increases oxidative stress by accumulating ROS, depleting antioxidant defenses, and causing cellular damage. This oxidative stress is linked to diabetes development [7]. The combination of the nicotinamide/STZ model and HFD is valuable for studying the metabolic abnormalities associated with T2DM, including abnormal plasma glucose and insulin levels, disrupted lipid metabolism, the generation of oxidative stress, and liver dysfunction. This model also enables the investigation of therapeutic approaches designed to enhance β-cell function and improve insulin sensitivity [8].

Electrolyzed water undergoes redox reactions that yield a distinct cathodic water composition, characterized by elevated concentrations of hydroxide ions (OH⁻), a pH exceeding 7, a negative oxidation-reduction potential (ORP), reduced dissolved oxygen levels, and increased soluble hydrogen. This specialized water, referred to as alkaline reduced water (ARW), possesses strong reducing properties and is free from residual chlorine [9]. ARW has shown potential in improving metabolic health by influencing key markers associated with diabetes, obesity, and dyslipidemia [10]. Studies on both animal models and humans suggest that ARW can lower blood sugar, triglycerides, and cholesterol levels, potentially due to its antioxidant properties. These effects are believed to arise from its ability to scavenge free radicals and enhance lipid metabolism. ARW also supports insulin production by improving pancreatic function and maintaining body alkalinity [11,12,13,14]. Additionally, ARW has demonstrated anti-obesity effects by reducing fat accumulation, enhancing adiponectin expression, and regulating lipid metabolism. Furthermore, ARW may help mitigate oxidative stress, which is linked to both diabetes and obesity, while improving hydration status during physical activity [15]. Furthermore, with a pH range of 8 to 10, ARW appears to favorably impact conditions such as diabetes and obesity while amplifying the benefits of lifestyle interventions, including exercise. ARW is proposed to lower oxidative stress and inflammatory markers, neutralize reactive oxygen species, and promote optimal hydration and acid-base balance, which may help mitigate electrolyte imbalances and enhance metabolic processes [16]. However, despite these reported benefits, the impact of ARW on glucose and lipid metabolism in both healthy and diabetic conditions is still not well understood. This study seeks to determine whether supplementing drinking water with ARW can enhance glucose and lipid metabolism while also reducing oxidative damage in non-diabetic rats and in diabetic rats induced by nicotinamide/STZ combined with an HFD.

## 2. Materials and Methods

### 2.1. Alkaline Reduced Water (ARW), Chemicals, and Reagents

ARW, with a measured pH range of approximately 10.0–10.5 (PHC2701 electrode; Meter Lab Radiometer Copenhagen, København, Denmark) and an ORW at −250 mV to −400 mV (GO51 electrode; Innovative Sensors, Inc., Las Vegas, NV, USA), was produced by an electrolyzed water generator (Kiranos AI-2200, TOYO Autech System, Inc., Osaka, Japan). Distilled water was obtained using a Milli-Q SP reagent water system (Millipore, Billerica, MA, USA). Chemicals and reagents were sourced from Sigma Chemical (St. Louis, MO, USA), Yakuri pure chemicals Co., Ltd. (Osaka, Japan), Panreac Química SLU (Barcelona, Spain), Wako Co. (Osaka, Japan), Roche Diagnostics Co. (Mannheim, Germany), and Merck (Darmstadt, Germany), unless specified otherwise.

### 2.2. Animal Experiment

Four-week-old male Wistar rats were sourced from the Laboratory Animal Center at the National Taiwan University College of Medicine. The rats were housed individually. Following a 5-week acclimatization phase, an oral glucose tolerance test (OGTT) was conducted to validate the successful induction of diabetes via the nicotinamide/STZ method, as detailed in previous studies [17]. The rats were randomly allocated into four groups: non-diabetic rats administered distilled water, non-diabetic rats given ARW, diabetic rats receiving distilled water, and diabetic rats receiving ARW. Each group included 10 rats, all maintained on a high-fat diet (HFD) (Table 1) with ad libitum access to either distilled water or ARW for 6 weeks. A freshly prepared HFD was provided to the rats daily. Prior to euthanasia, a repeat OGTT was carried out, and the area under the curve (AUC) was calculated using established methodologies from prior research [18]. On the euthanasia day, the rats were sedated with isoflurane, and samples of blood, liver, kidney, intestinal tissue, and feces were collected for subsequent analysis. The treatments, measurements, and cage locations were assigned randomly.

### 2.3. Determination of Plasma Biochemistry, Lipid Profile, TBARS Levels, and Antioxidant Enzyme Activities

Plasma levels of glucose, insulin, fructosamine, leptin, lactate, glutamic oxaloacetic transaminase (GOT), glutamic pyruvic transaminase (GPT), uric acid, creatinine, total cholesterol, and triglycerides were assessed using commercially available assay kits from various suppliers, including Sigma Co. (St. Louis, MO, USA), Assay Designs, Inc. (Ann Arbor, MI, USA), BioVision Research Products (Mountain View, CA, USA), Audit Diagnostics (Cork, Ireland), Mercodia AB Inc. (Uppsala, Sweden), and Hospitex Diagnostics LP (League City, TX, USA). The concentrations of lipoproteins in the plasma and lipid content in the liver and fecal samples were determined using techniques previously described in the literature [19,20]. The contents of fecal bile acid were analyzed using a Bile acid kit (RANDOX Laborator Ltd., Crumlin, UK) according to the supplier’s instructions. The levels of thiobarbituric acid reactive substances (TBARS) were measured through the reaction of thiobarbituric acid with lipid peroxide products, specifically malondialdehyde (MDA), in the plasma and liver lysate samples. The methods of assessing glutathione (GSH), glutathione disulfide (GSSG), glutathione peroxidase (GSH Px), glutathione reductase (GSH Rd), glutathione-S-transferase (GSH-S-T), catalase, and superoxide dismutase (SOD) followed the protocols detailed in previous studies [21,22,23,24,25,26].

### 2.4. Measurement of the Activities of G-6-Pase, Hexokinase, G-6-P DeHase, and Intestinal Disaccharidase

The enzymatic activities of hexokinase, glucose-6-phosphatase (G-6-Pase), and glucose-6-phosphate dehydrogenase (G-6-P DeHase) in the cytosolic fraction were evaluated according to established protocols [27]. Fresh segments of the distal and proximal small intestine were isolated, and the mucosal tissues were collected and weighed. The activities of lactase, sucrase, and maltase in both segments were measured using methods described in prior studies [28,29].

### 2.5. Statistical Analysis

The experimental data were processed using SPSS/PC version 28 for statistical analysis. Independent-sample *t*-tests were utilized to compare each experimental group with its corresponding control group. Additionally, a two-way analysis of variance (ANOVA) was conducted to evaluate the effects of diabetes mellitus (DM) and alkaline reduced water (ARW) consumption, focusing on both the individual contributions of these factors and their interactions.

## 3. Results

### 3.1. Influence of ARW Supplementation on Modulating Glucose Metabolism

**Figure 1:**
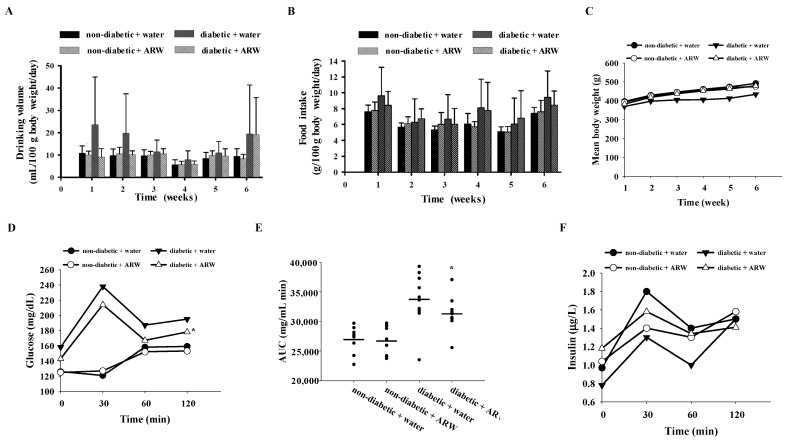
Changes of (**A**) drinking volume, (**B**) food intake, (**C**) mean body weight, (**D**) plasma glucose, (**E**) area under the curve and (**F**) insulin levels during OGTT for non-diabetic and diabetic rats drinking ARW for 6 weeks. Results are shown as mean ± standard deviation (n = 10 rats per group). * *p* < 0.05 signifies a significant difference when compared to the corresponding water control, determined by independent-sample *t*-test.

The average drinking volume, food intake, and gain of body weight of the rats did not differ significantly among the four groups throughout the experiment (Figure 1A–C). The variations in drinking volume may be attributed to differences in the extent of polydipsia recovery among individual rats following nicotinamide/STZ injection. The results from the OGTT, analyzed through two-way ANOVA, indicated that plasma glucose levels were significantly elevated in the diabetic group across all time points compared to the non-diabetic group (Figure 1D). The diabetic rats that received ARW supplementation exhibited notably lower plasma glucose levels than those in the water control group (Figure 1D). Furthermore, ARW supplementation led to a significant decrease in the plasma glucose AUC for diabetic rats (Figure 1E). However, there were no significant differences in plasma insulin levels among the four groups at any time point measured (Figure 1F). Notably, non-diabetic rats generally have a more robust and rapid insulin response to oral glucose, which may moderate the peak glucose levels but delay the timing of the peak as the body processes glucose efficiently.

The concentrations of fasting plasma glucose were raised, while insulin concentrations were reduced in diabetic rats compared to non-diabetic rats. Importantly, rats receiving ARW supplementation exhibited significantly lower fasting plasma glucose and insulin levels compared to those given distilled water. Conversely, the plasma leptin levels in diabetic rats were significantly lower than those in non-diabetic rats, and ARW consumption significantly reduced plasma leptin levels in non-diabetic rats. No notable differences in fructosamine levels were found among the groups (Table 2).

**Figure 2:**
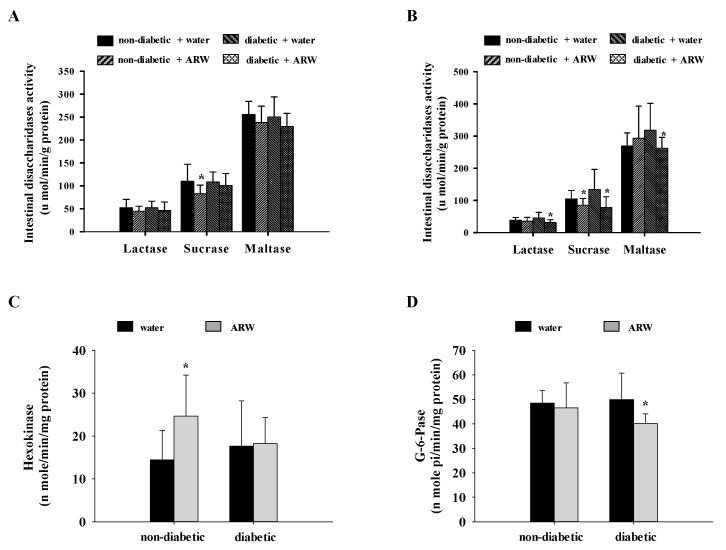
The (**A**) proximal region and (**B**) distal intestinal disaccharidases, and hepatic (**C**) hexokinase, and (**D**) G-6-Pase activities of non-diabetic and diabetic rats drinking alkaline reduced water for 6 weeks. Results are shown as mean ± standard deviation (n = 10 rats per group). * *p* < 0.05 signifies a significant difference when compared to the corresponding water control, determined by independent-sample *t*-test.

In non-diabetic rats consuming ARW, the activities of sucrase in both small intestinal segments were reduced compared to those consuming distilled water. Similarly, in diabetic rats consuming ARW, the activities of lactase, sucrase, and maltase in the distal small intestinal segment were significantly reduced compared to the distilled water group (Figure 2A,B). Furthermore, the activity of hexokinase in non-diabetic rats consuming ARW was significantly higher than in those receiving distilled water (Figure 2C). In diabetic rats supplemented with ARW, the activity of G-6-Pase was downregulated compared to the distilled water group (Figure 2D). Overall, the modulation of insulin sensitivity, disaccharide digestion, and glucose metabolism may be potential mechanisms underlying the hypoglycemic activity of ARW.

### 3.2. Effect of ARW Supplementation on Improving Lipid Metabolism

The interaction between diabetes and the consumption of ARW significantly influenced the plasma lipid profile. In non-diabetic rats, ARW consumption led to decreases in the concentrations of total cholesterol, LDL-C + VLDL-C, triglycerides, HDL-TG, and LDL-TG + VLDL-TG, as well as reductions in the cholesterol/HDL-C and (LDL-C + VLDL-C)/HDL-C ratios. In diabetic rats, ARW consumption resulted in downregulated HDL-C concentration and HDL-TG/(LDL-TG + VLDL-TG) ratio, while LDL-TG + VLDL-TG concentration was upregulated (Table 3).

Regarding the hepatic lipid profile, the cholesterol content per gram of liver and total liver weight were significantly greater in the diabetic group compared to the non-diabetic group. In contrast, non-diabetic rats that consumed ARW showed an increase in hepatic cholesterol content. However, no significant differences in hepatic triglyceride levels were noted across the groups (Table 4). While there was a trend toward lower fecal cholesterol content in the diabetic group, this difference did not reach statistical significance. Non-diabetic rats that received ARW showed significantly reduced cholesterol levels compared to those given distilled water. The interaction between diabetes and ARW consumption also had a significant impact on triglyceride content per gram of feces and total daily feces. ARW consumption led to increased fecal triglyceride levels, with diabetic rats exhibiting significantly higher triglyceride levels than non-diabetic rats. Furthermore, diabetic rats supplemented with ARW had significantly elevated triglyceride levels both per gram of feces and in total daily feces compared to those receiving distilled water. Additionally, fecal bile acid content was significantly lower in diabetic rats compared to non-diabetic rats, while ARW consumption significantly boosted fecal bile acid content in both non-diabetic and diabetic rats (Table 4). These findings suggest that ARW may have beneficial effects in reducing hyperlipidemia, likely by enhancing lipid metabolism and excretion. Notably, in non-diabetic rats supplemented with ARW, the cholesterol content per unit of liver tissue significantly increased. While the total hepatic cholesterol levels did not show a notable difference, fecal cholesterol excretion was markedly reduced. This reduction in fecal cholesterol excretion is speculated to contribute to the accumulation of cholesterol in the liver.

### 3.3. Antioxidative Potential of ARW Supplementation

The interaction between diabetes and the consumption of ARW did not significantly affect hepatic and nephric TBARS contents, as no statistical differences were observed among the experimental groups compared to their corresponding control groups (Table 5). However, this interaction significantly influenced hepatic GSH content. Non-diabetic rats consuming ARW exhibited significantly higher hepatic GSH levels than those consuming distilled water. Conversely, diabetic rats supplemented with ARW showed significantly reduced hepatic GSH levels compared to those receiving distilled water. Regarding GSSG content, diabetic rats had significantly lower hepatic GSSG levels compared to non-diabetic rats, but no statistical differences were observed among the experimental groups compared to their respective control groups. The GSH/GSSG ratio was notably elevated in diabetic rats compared to non-diabetic rats. The interaction between diabetes and ARW also significantly affected GSH Px activity. Non-diabetic rats consuming ARW displayed a substantial increase in hepatic GSH Px activity. Additionally, ARW consumption significantly enhanced GSH Rd activity in both non-diabetic and diabetic rats. Diabetic rats demonstrated a significant reduction in hepatic GSH-S-T activity compared to non-diabetic rats, although ARW consumption did not significantly impact GSH-S-T activity, catalase, or SOD. Non-diabetic rats consuming ARW exhibited significantly higher G-6-P DeHase activity compared to those receiving distilled water (Table 5).

To further evaluate the effects of ARW on liver and kidney function, biochemical analyses were conducted (Table 6). Non-diabetic rats consuming ARW showed increased GOT activity compared to the control group, which showed no significant differences in GPT levels. After six weeks of ARW consumption, uric acid levels showed no significant differences among the groups. Non-diabetic rats consuming ARW had significantly lower creatinine levels than their control group, whereas diabetic rats supplemented with ARW had higher creatinine levels compared to their respective control group.

## 4. Discussion

The promise of ARW as a treatment option for diabetes management has gained significant interest due to its reported anti-diabetic, anti-obesity, and antioxidant properties [10,16,30]. Nonetheless, how ARW influences glucose and lipid metabolism, along with oxidative stress in both non-diabetic and diabetic states, has not been thoroughly examined. This study revealed that ARW supplementation enhanced glucose and lipid metabolism, along with antioxidant capacity, though the results for non-diabetic and diabetic rats consuming ARW were not completely consistent.

Previous research has demonstrated that ARW, when administered as drinking water, can significantly lower blood glucose levels and enhance glucose tolerance in genetically diabetic mice and STZ-induced diabetic mice. Consistent with our findings, ARW did not have a significant effect on insulin levels in STZ-induced diabetic mice, but it did lead to an increase in insulin levels in db/db mice. Accordingly, the improved blood glucose control associated with ARW may be linked to enhanced insulin sensitivity and secretion [31]. In this study, non-diabetic rats consuming ARW exhibited a marked decrease in intestinal sucrase activity. Diabetic rats consuming ARW similarly showed reduced sucrase activity in the distal segment. Given that sucrase plays a crucial role in hydrolyzing disaccharides into absorbable monosaccharides, the diminished activity observed in diabetic rats may contribute to lower plasma glucose levels by delaying carbohydrate absorption.

Further investigations into diabetes management using Otsuka Long-Evans Tokushima Fatty rats revealed that ARW consumption over 12 weeks significantly reduced the concentrations of glucose, total cholesterol, and triglyceride [11]. Our findings demonstrated that while ARW supplementation for 6 weeks reduced total cholesterol and triglyceride levels in non-diabetic rats, no significant changes were observed in diabetic rats. This suggests that the duration of ARW supplementation may be critical to improving plasma lipid profiles in diabetic conditions. Additionally, variability in dietary cholesterol content across studies may explain discrepancies in findings regarding triglyceride levels. In an HFD-induced obesity model, mice receiving ARW displayed significant reductions in adiposity, weight gain, and fat accumulation in both epididymal and liver tissues [32]. These results, along with our findings, suggest that ARW consumption not only ameliorates hyperlipidemia induced by HFD but also mitigates body fat accumulation, highlighting its beneficial role in obesity management.

Clinical studies provide additional evidence for the potential therapeutic advantages of ARW. A randomized, double-blind controlled trial that included 30 individuals diagnosed with T2DM found that participants consuming ARW exhibited lower fasting blood glucose levels post-treatment than those receiving mineral water, despite the differences not reaching statistical significance [33]. Additionally, a cross-sectional comparative study on postmenopausal women with metabolic syndrome indicated that ARW drinkers had lower fasting plasma glucose levels and improved triglyceride-to-HDL ratios, underscoring the potential health benefits associated with regular ARW consumption [34].

ARW has shown promise in enhancing antioxidant capacity by modulating oxidative stress [10,16,30]. In this study, ARW consumption significantly elevated hepatic GSH and GSH Px activities, likely due to enhanced GSH Rd activity, which relies on NADPH to regenerate active GSH. Increased G-6-P DeHase activity in non-diabetic rats further supports improved NADPH availability and efficient GSH recovery. Although hepatic TBARS levels were unchanged, the elevated GSH/GSSG ratio and enhanced antioxidant enzyme activities suggest a compensatory response to oxidative stress, particularly in diabetic rats. These results align with previous studies showing that ARW reduces ROS and nitric oxide levels while increasing GSH Px activity after intense exercise [35]. Biochemical analyses revealed that ARW improved kidney function markers in non-diabetic rats, including increased GOT activity and reduced creatinine levels, without affecting uric acid. However, diabetic rats showed increased creatinine levels with ARW supplementation, indicating potential risks that require further investigation. In summary, ARW enhances enzymatic antioxidant defenses and shows protective effects on kidney function in non-diabetic contexts, although its benefits in diabetic models remain unclear. These findings highlight ARW’s potential as a dietary supplement to improve antioxidant capacity, warranting further research on its long-term impacts and mechanisms.

## 5. Conclusions

This study comprehensively explored and compared the effects of ARW on glucose and lipid metabolism, along with antioxidant capacity, in both non-diabetic and diabetic rats. The findings indicate that ARW supplementation holds significant promise for positively impacting these metabolic processes and enhancing antioxidant defenses. These results highlight the potential preventive and therapeutic benefits of ARW in managing metabolic disorders. Nevertheless, further research is necessary to clarify the cellular and molecular mechanisms driving these effects, which will facilitate more targeted applications to diabetes and related conditions.

## Figures and Tables

**Table 1:** High-fat diet (HFD) composition.

Ingredient	HFD (Weight, %)
Casein	20.0
Lard	10.0
AIN-76 Vitamin mixture	1.0
AIN-76 Mineral mixture	4.0
Cholesterol	0.5
Cholic acid	0.1
Choline chloride	0.2
Cellulose	5.0
Corn starch	59.2
Total	100

The caloric density of the HFD is approximately 4.07 kcal/g.

**Table 2:** Fasting plasma glucose, insulin, leptin, and fructosamine levels of non-diabetic and diabetic rats drinking distilled water or ARW for 6 weeks.

	Non-Diabetic		Diabetic		Two-Way ANOVA		
	Water	ARW	Water	ARW	DM	ARW	DM × ARW
Glucose (mg/dL)	177.2 ± 10.0	166.2 ± 11.8 *	206.1 ± 36.2	175.5 ± 14.4 *	0.0090 **	0.0050 **	NS
Insulin (μg/L)	3.00 ± 0.11	1.91 ± 0.68 *	1.38 ± 0.76	1.09 ± 0.43	0.0001 **	0.0030 **	NS
Leptin (ng/mL)	4.96 ± 0.55	4.32 ± 0.86 *	3.24 ± 1.32	3.66 ± 1.60	0.0040 **	NS	NS
Fructosamine (μmol/L)	175.2 ± 62.9	144.8 ± 66.1	199.6 ± 63.8	169.6 ± 64.8	NS	NS	NS

Results are shown as mean ± standard deviation (n = 10 rats per group). * *p* < 0.05 signifies a significant difference when compared to the corresponding water control, determined by independent-sample *t*-test. ** The mean difference is significant at the 0.01 level. DM: diabetes; ARW: alkaline reduced water; DM × ARW: diabetes × alkaline reduced water, by two-way ANOVA. NS: Not significant.

**Table 3:** Plasma lipid profile of non-diabetic and diabetic rats drinking distilled water or ARW for 6 weeks.

	Non-Diabetic		Diabetic		Two-Way ANOVA		
	Water	ARW	Water	ARW	DM	ARW	DM × ARW
Total cholesterol (TC) (mg/dL)	113.7 ± 24.9	92.3 ± 17.8 *	125.9 ± 41.4	131.1 ± 37.2	NS	NS	0.024
HDL-C (mg/dL)	22.7 ± 5.5	24.1 ± 5.5	34.6 ± 11.2	27.2 ± 7.8 *	0.024	NS	0.017
LDL + VLDL-C (mg/dL)	91.0 ± 25.0	68.2 ± 19.1 *	93.6 ± 36.0	116.7 ± 46.5	0.030	NS	0.050
TC/HDL-C	5.25 ± 1.63	4.03 ± 1.26 *	4.00 ± 1.18	5.34 ± 1.68	NS	NS	0.015
(LDL + VLDL-C)/HDL-C	4.25 ± 1.63	3.03 ± 1.26 *	3.00 ± 1.18	4.34 ± 1.68	NS	NS	0.015
Triglyceride (TG) (mg/dL)	101.8 ± 22.8	77.8 ± 17.8 *	90.1 ± 25.7	106.5 ± 30.9	NS	NS	0.020
HDL-TG (mg/dL)	16.5 ± 3.3	13.9 ± 1.0 *	14.8 ± 0.9	16.9 ± 3.4	NS	NS	0.010 **
LDL + VLDL-TG (mg/dL)	85.3 ± 22.3	62.5 ± 19.9 *	63.5 ± 15.8	85.3 ± 30.7 *	NS	NS	0.008 **
HDL-TG/(LDL + VLDL-TG)	0.21 ± 0.09	0.27 ± 0.05	0.27 ± 0.08	0.21 ± 0.20 *	NS	NS	NS

Results are shown as mean ± standard deviation (n = 10 rats per group). * *p* < 0.05 signifies a significant difference when compared to the corresponding water control, determined by independent-sample *t*-test. ** The mean difference is significant at the 0.01 level. DM: diabetes; ARW: alkaline reduced water; DM × ARW: diabetes × alkaline reduced water, by two-way ANOVA. NS: Not significant.

**Table 4:** Hepatic and fecal lipid profile of non-diabetic and diabetic rats drinking distilled water or ARW for 6 weeks.

	Non-Diabetic		Diabetic		Two-Way ANOVA		
	Water	ARW	Water	ARW	DM	ARW	DM × ARW
Hepatic total cholesterol (mg/g)	84.9 ± 16.7	109.8 ± 22.2 *	127.6 ± 28.3	126.3 ± 20.6	0.0001 **	NS	NS
Hepatic total cholesterol (g/liver)	1.7 ± 0.7	2.2 ± 0.6	2.6 ± 1.0	3.0 ± 0.6	0.0020 **	NS	NS
Hepatic triglyceride (mg/g)	109.7 ± 30.7	124.8 ± 32.0	126.2 ± 30.4	122.7 ± 21.0	NS	NS	NS
Hepatic triglyceride (g/liver)	2.2 ± 0.9	2.5 ± 0.7	2.5 ± 0.6	2.9 ± 0.5	NS	NS	NS
Fecal total cholesterol (mg/g)	24.7 ± 3.2	20.3 ± 3.9 *	15.4 ± 5.5	15.3 ± 4.0	0.0001 **	NS	NS
Fecal total cholesterol (mg/day)	45.5 ± 8.4	35.7 ± 9.6 *	33.5 ± 13.0	33.6 ± 9.0	NS	NS	NS
Fecal triglyceride (mg/g)	2.1 ± 0.5	2.3 ± 0.4	2.8 ± 0.9	6.7 ± 2.5 *	0.0001 **	0.0001 **	0.0001 **
Fecal triglyceride (mg/day)	3.9 ± 0.9	4.1 ± 1.3	6.7 ± 4.5	11.4 ± 3.4 *	0.0001 **	0.0220	0.0350 **
Fecal bile acid (μmol/g)	43.8 ± 4.0	44.3 ± 4.8	38.3 ± 6.7	40.1 ± 7.2	0.0190 **	NS	NS
Fecal bile acid (μmol/day)	79.7 ± 9.7	82.6 ± 8.0	67.9 ±17.6	83.6 ± 14.3 *	NS	0.0470	NS

Results are shown as mean ± standard deviation (n = 10 rats per group). * *p* < 0.05 signifies a significant difference when compared to the corresponding water control, determined by independent-sample *t*-test. ** The mean difference is significant at the 0.01 level. DM: diabetes; ARW: alkaline reduced water; DM × ARW: diabetes × alkaline reduced water, by two-way ANOVA. NS: Not significant.

**Table 5:** Hepatic and nephric TBARS value and antioxidant enzyme activity of non-diabetic and diabetic rats drinking distilled water or ARW for 6 weeks.

	Non-Diabetic		Diabetic		Two-Way ANOVA		
	Water	ARW	Water	ARW	DM	ARW	DM × ARW
Hepatic TBARS (n mole/g)	24.9 ± 3.4	24.9 ± 5.2	23.0 ± 3.2	25.4 ± 4.9	NS	NS	NS
Nephric TBARS (nmole/g)	26.6 ± 10.2	30.3 ± 11.2	22.3 ± 7.7	20.9 ± 8.6	0.034	NS	NS
Hepatic GSH (nmol/g liver)	1.7 ± 0.8	2.3 ± 0.4 *	2.5 ± 0.5	1.8 ± 0.7 *	NS	NS	0.007 **
Hepatic GSSG (nmol/g liver)	0.6 ± 0.2	0.5 ± 0.1	0.4 ± 0.2	0.3 ± 0.1	0.001 **	NS	NS
Hepatic GSH/GSSG ratio	3.6 ± 3.5	4.0 ± 1.4	7.8 ± 6.5	6.5 ± 3.6	0.038	NS	NS
Hepatic GSH Px (nmol NADPH oxidized/min/mg protein)	66.8 ± 9.9	82.5 ± 10.8 *	77.5 ± 12.8	75.7 ± 10.2	NS	0.048	0.029
Hepatic GSH Rd (nmol NADPH oxidized/min/mg protein)	13.1 ± 2.1	16.3 ± 3.5 *	13.8 ± 2.9	15.7 ± 4.0	NS	0.025	NS
Hepatic GSH-S-T (nmol CDNB-GSH conjugate formed oxidized/min/mg protein)	300.9 ± 63.5	306.8 ± 37.0	268.1 ± 49.0	267.0 ± 50.9	0.044	NS	NS
Hepatic Catalase (K^−2^/mg protein)	38.1 ± 14.3	43.0 ± 19.8	38.6 ± 12.5	28.2 ± 8.5	NS	NS	NS
Hepatic SOD (U/mg protein)	74.1 ± 5.9	72.1 ± 7.7	63.0 ± 16.6	70.4 ± 6.5	NS	NS	NS
Hepatic G-6-P DeHase (nmole/min/mg protein)	12.9 ± 6.2	19.5 ± 7.4 *	15.4 ± 8.4	18.3 ± 4.9	NS	0.045	NS

Results are shown as mean ± standard deviation (n = 10 rats per group). * *p* < 0.05 signifies a significant difference when compared to the corresponding water control, determined by independent-sample *t*-test. ** The mean difference is significant at the 0.01 level. DM: diabetes; ARW: alkaline reduced water; DM × ARW: diabetes × alkaline reduced water, by two-way ANOVA. NS: Not significant.

**Table 6:** Plasma biochemical profile of non-diabetic and diabetic rats drinking distilled water or ARW for 6 weeks.

	Non-Diabetic		Diabetic		Two-Way ANOVA		
	Water	ARW	Water	ARW	DM	ARW	DM × ARW
GOT (U/L)	44.39 ± 13.29	60.64 ± 23.63 *	58.98 ± 24.74	50.61 ± 9.68	NS	NS	NS
GPT (U/L)	20.03 ± 6.85	27.32 ± 12.77	28.64 ± 11.53	24.20 ± 3.95	NS	NS	NS
Uric acid (mg/dL)	1.28 ± 0.27	1.25 ± 1.89	1.42 ± 0.47	1.31 ± 0.39	NS	NS	NS
Creatinine (mg/dL)	0.62 ± 0.04	0.53 ± 0.05 *	0.55 ± 0.07	0.65 ± 0.10 *	NS	NS	0.0001 **

Results are shown as mean ± standard deviation (n = 10 rats per group). * *p* < 0.05 signifies a significant difference when compared to the corresponding water control, determined by independent-sample *t*-test. ** The mean difference is significant at the 0.01 level. DM: diabetes; ARW: alkaline reduced water; DM × ARW: diabetes × alkaline reduced water, by two-way ANOVA. NS: Not significant.

## Data Availability

The original contributions presented in the study are included in the article.
